# Sensitivity of Rodent Microglia to Kynurenines in Models of Epilepsy and Inflammation In Vivo and In Vitro: Microglia Activation Is Inhibited by Kynurenic Acid and the Synthetic Analogue SZR104

**DOI:** 10.3390/ijms21239333

**Published:** 2020-12-07

**Authors:** Noémi Lajkó, Diana Kata, Melinda Szabó, Adrienne Mátyás, Karolina Dulka, Imre Földesi, Ferenc Fülöp, Karoly Gulya, László Vécsei, András Mihály

**Affiliations:** 1 Department of Cell Biology and Molecular Medicine, University of Szeged, 6720 Szeged, Hungary; lajko.noemi@med.u-szeged.hu (N.L.); szabo.melinda.1@med.u-szeged.hu (M.S.); dulka.karolina@med.u-szeged.hu (K.D.); gulyak@bio.u-szeged.hu (K.G.); 2 Department of Laboratory Medicine, University of Szeged, 6720 Szeged, Hungary; kata.diana@med.u-szeged.hu (D.K.); foldesi.imre@med.u-szeged.hu (I.F.); 3 Department of Anatomy, University of Szeged, 6720 Szeged, Hungary; matyas.adrienne@med.u-szeged.hu; 4 Institute of Pharmaceutical Chemistry and Research Group for Stereochemistry, Hungarian Academy of Sciences, University of Szeged, 6720 Szeged, Hungary; fulop@pharm.u-szeged.hu; 5 Department of Neurology, University of Szeged, 6720 Szeged, Hungary; vecsei.laszlo@med.u-szeged.hu; 6 Department of Neurology, Interdisciplinary Excellence Centre, University of Szeged, 6720 Szeged, Hungary; 7 Department of Anatomy, Faculty of Medicine and Health Sciences, Carl von Ossietzky University Oldenburg, 26129 Oldenburg, Germany

**Keywords:** epilepsy, inflammation, kynurenic acid, lipopolysaccharide, microglia, pilocarpine, secondary culture, SZR104

## Abstract

Kynurenic acid is an endogenous modulator of ionotropic glutamate receptors and a suppressor of the immune system. Since glutamate and microglia are important in the pathogenesis of epilepsy, we investigated the possible action of the synthetic kynurenic acid analogue, SZR104, in epileptic mice and the action of kynurenic acid and SZR104 on the phagocytotic activity of cultured microglia cells. Pilocarpine epilepsy was used to test the effects of SZR104 on morphological microglia transformation, as evaluated through ionized calcium-binding adaptor molecule 1 (Iba1) immunohistochemistry. Microglia-enriched rat secondary cultures were used to investigate phagocytosis of fluorescent microbeads and Iba1 protein synthesis in control and lipopolysaccharide-challenged cultures. SZR104 inhibited microglia transformation following status epilepticus. Kynurenic acid and SZR104 inhibited lipopolysaccharide-stimulated phagocytotic activity of microglia cells. Although kynurenic acid and its analogues proved to be glutamate receptor antagonists, their immunosuppressive action was dominant in epilepsy. The inhibition of phagocytosis in vitro raised the possibility of the inhibition of genes encoding inflammatory cytokines in microglial cells.

## 1. Introduction

Pharmacological experiments proved that endogenous kynurenic acid (4-hydroxyquinolin-2-carboxylic acid, C_10_H_7_NO_3_; KYNA) exerted a neuroprotective role in different inflammatory/neurodegenerative central nervous system (CNS) disorders [1,2]. KYNA is an endogenous antagonist of the N-methyl-D-aspartic acid (NMDA) receptor, and therefore, it may protect cells against the cytotoxic actions of glutamic acid [1,2]. However, exogenous KYNA is not able to cross the blood–brain barrier (BBB); therefore, efforts are being made to synthesize KYNA analogues which penetrate the BBB, enter the brain and, supposedly, can be used in the pharmacological treatment of some neurodegenerative disorders [3]. One of these KYNA analogues, C_19_H_26_N_4_O_3_ or N-(2-(dimethylamino)ethyl)-3-(morpholinomethyl)-4-hydroxyquinoline-2-carboxamide (SZR104), has been applied successfully in pentylenetetrazol (PTZ) seizures and significantly decreased the seizure-evoked field potentials [4]. These experiments were performed on rats in urethane anesthesia and investigated the electrophysiological effects of SZR104 pretreatment on PTZ convulsions [4]. In the present experiments, we aimed to study the pharmacological effects of SZR104 in the non-anesthetized, epileptic brain. Generalized epilepsy was induced in mice with pilocarpine (PILO), since our previous experiments proved that acute, generalized convulsive seizures significantly increase the brain extracellular concentration of glutamic acid [5]. It was also proven that the awake epilepsy model was useful for the pharmacological evaluation of some antiepileptic drugs [6].

KYNA also affects the immune system through the regulation and modulation of the cell cycle of effector immune cells [7,8]. Microglial cells are the effector immune cells of the brain, participating in every neuropathological event [9,10,11]. Microglia are activated and transformed by neurotransmitters (e.g., glutamate acting on microglial NMDA receptors [12]), damage-associated stress signals, cytokines, colony-stimulating factors and free radicals [13,14,15]. In neuropathology, this disease-associated phenotype is called reactive microglia [9,10,13]. Reactive microglia cells display significant morphological transformations: they increase in size and develop a large number of cytoplasmic processes which help migration and phagocytosis [9,16]. Disease-associated microglia not only protects but also damages and kills neurons [11,13]. Therefore, we evaluated the microglia effects of SZR104 in PILO-induced mouse epilepsy. We evaluated the proliferation tendency (number) and the size of hippocampal microglia cells with the help of ionized calcium-binding adaptor molecule 1 (Iba1) immunohistochemistry [17]. The receptors and signaling mechanisms of microglia cells regulate the expression of their genome and help their adaptation to pathogenic environmental stimuli through the secretion of cytokines [13,18]. We measured the blood levels of the cytokine interleukin-6 (IL-6) in order to track the functional alterations of the microglia, in vivo [18].

The experimental investigation of the pharmacology of microglial phagocytosis in vivo is very difficult [19]; therefore, we performed in vitro experiments using microglia-enriched forebrain cell cultures [20,21]. Our previous experiments proved that microglial cell cultures are suitable for the evaluation of the effects of anti-inflammatory compounds, and the in vitro lipopolysaccharide (LPS) challenge can be successfully used [21,22] in experimental pharmacological investigations [20,22]. In the present experiments, we evaluated the in vitro microglial phagocytosis induced by LPS and tested the pharmacological effects of SZR104 and KYNA by counting the number of internalized fluorescent microbeads [21,22]. The Iba1 expression of the microglia cells has been measured with Western blotting.

We observed that the concentration of IL-6 increased significantly in the venous blood of the epileptic mice. The KYNA analogue SZR104 significantly inhibited the in vivo transformation of hippocampal microglia cells from the resting into the activated/reactive forms. SZR104 also decreased the blood IL-6 level. However, the SZR104 pretreatment failed to reduce the motor hyperactivity and the status epilepticus in mice. We found that both KYNA and SZR104 significantly inhibited the in vitro phagocytosis of fluorescent microbeads in microglia cells induced by LPS treatment.

## 2. Results

### 2.1. Seizure Symptoms

PILO-treated mice displayed motor hyperactivity beginning 10–15 min after the intraperitoneal (i.p.) injections. The first symptoms were tremor of the limbs, movement automatisms and wild running. Then, the animal fell on its side, displaying frequent limb flexions and trunk extension (tonic-clonic seizure). After a transient (6–8 min) recovery, the motor symptoms were repeated—often for 80–120 min. Not every animal displayed generalized tonic-clonic seizure; 35% of the mice were displaying only tremor, automatisms and wild running episodes lasting for 60–90 min. The animals which were pretreated with SZR104 (SZR104 and PILO-treated animals) presented similar symptoms; the latency of the motor symptoms and tonic-clonic convulsions was not delayed nor prevented by the KYNA analogue SZR104. The convulsing animals injected only with PILO suffered a 30% mortality rate. The combined treatment with 380 mg/kg SZR104 and 190 mg/kg PILO did not decrease the mortality rate; on the contrary, this animal group had a mortality rate of 40%. The solvent of pilocarpine (0.9% NaCl) and the 380 mg/kg SZR104 injected alone did not cause symptoms and did not cause mortality. Animals were further investigated after 24 h, following the treatments (Table 1).

### 2.2. IL-6 Levels in Blood

**Figure 1:**
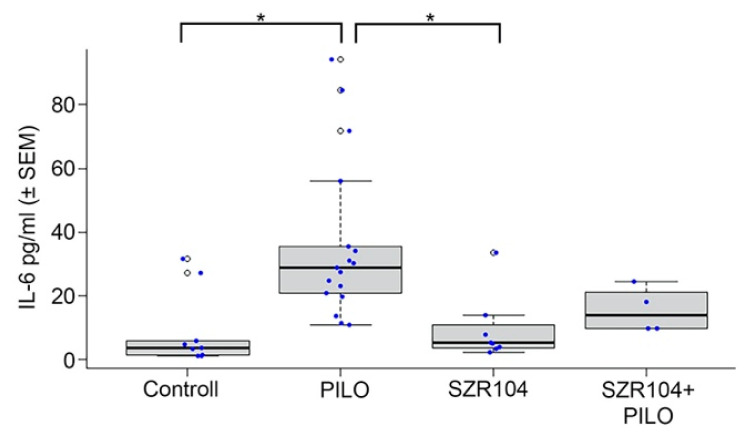
Interleukin-6 (IL-6) concentration (mean ± SEM) in the blood 24 h after pharmacological treatments. Statistically significant differences were detected between controls and PILO-treated and between pilocarpine(PILO)-treated and SZR104-treated animals (* *p* ≤ 0.05). SZR104-pretreated epileptic animals (SZR104 + PILO) displayed decreased IL-6 level, but the decrease was not statistically significant (blood IL-6 concentrations in pg/mL; blue dots display the number of measurements/blood samples).

Significantly increased IL-6 levels were detected in the PILO-treated mice (Figure 1). In the venous blood taken 24 h after the pharmacological treatments, SZR104 pretreatment decreased the elevated IL-6 level, but the decrease (the effect of SZR104) was not significant. The levels of IL-6 were also slightly higher in SZR104 + PILO-treated mice than those in the controls; however, none of the alterations were significant (Figure 1). SZR104 alone did not cause any alterations; the IL-6 values were similar to the physiological saline-injected controls (Figure 1).

### 2.3. Iba1 Immunohistochemistry

**Figure 2:**
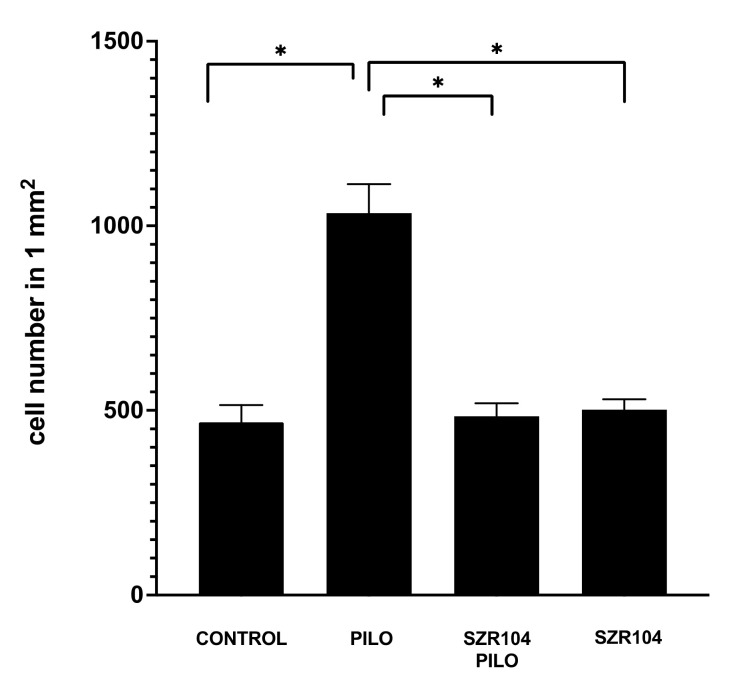
Analysis of ionized calcium-binding adaptor molecule 1 (Iba1)-positive cell counts in the entire area of the hippocampal formation of epileptic and control mice (*n =* 16; mean ± SEM). Statistical differences were detected between control, pilocarpine (PILO)-treated, SZR104 + PILO-treated and SZR104-treated animals (* *p* ≤ 0.05).

Control mice displayed normal microglia cells; Iba1-stained cells were evenly distributed in the hippocampus, their shape and distribution were similar to those of the resting microglia, the diameter of the cell body was approx. 6–7 μm and their ramified processes were thin and relatively short. The cells possessed 4–5 slender, 5–10-μm long processes (Figure 2). The PILO treatment induced a significant increase in the number of cells when counted after 24 h. The number of Iba1-positive cells in a 1-mm^2^ hippocampal area increased to 1700 per mm^2^ or above compared to the controls, in which we counted 500 cells or less in 1 mm^2^.

**Figure 3:**
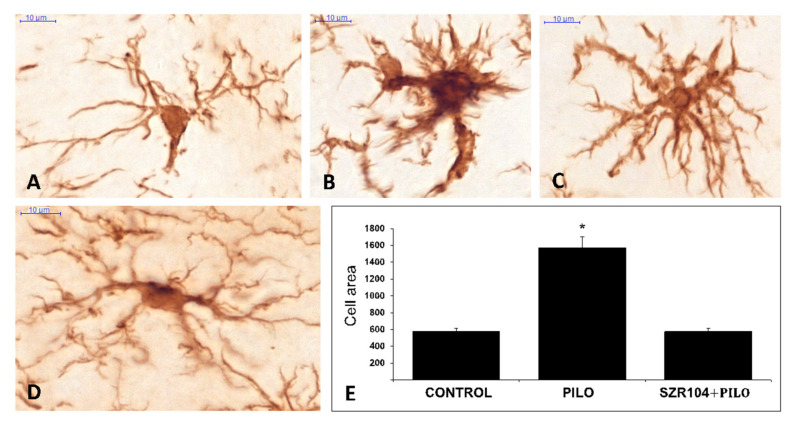
(**A**–**D**): ionized calcium-binding adaptor molecule 1 (Iba1)-stained microglia cells from control (**A**), pilocarpine(PILO)-treated (**B**,**C**) and SZR104 + PILO-treated (**D**) animals. Scale bars: 10 µm. (**E**): The average microglia cell areas (cell body and processes) in µm^2^ values on the y-axis (*n =* 10; mean ± SEM) in control, PILO-treated and SZR104 + PILO-treated animals (* *p* ≤ 0.05).

The size of the Iba1-stained cells was larger in PILO-treated mice; cell body diameters were 8–12 µm, and the cytoplasmic processes were thicker compared to resting microglia cells (Figure 3). The measured surface area of the cells increased significantly compared to the controls (Figure 3). While the control, untreated microglia cell areas were in the ≤500 μm^2^ range, the cells from epileptic animals were significantly larger (≤1500 μm^2^).

The SZR104 pretreatment of epileptic animals prevented the seizure-caused microglial alterations. Both the number of Iba1-positive cells and the surface area of the single cells remained close to the control values (Figure 3). When compared to the PILO-treated hippocampi, the microglia-inhibiting effects of SZR104 proved to be significant (Figure 2). The shape, size and ramification were similar to controls (Figure 3). SZR104 given alone did not cause alterations in the number and morphology of microglia cells—these cells were similar to the control cells (Figure 3).

### 2.4. Effect of KYNA and SZR104 on Microglia Phagocytosis and Iba1 Immunoreactivity

**Figure 4:**
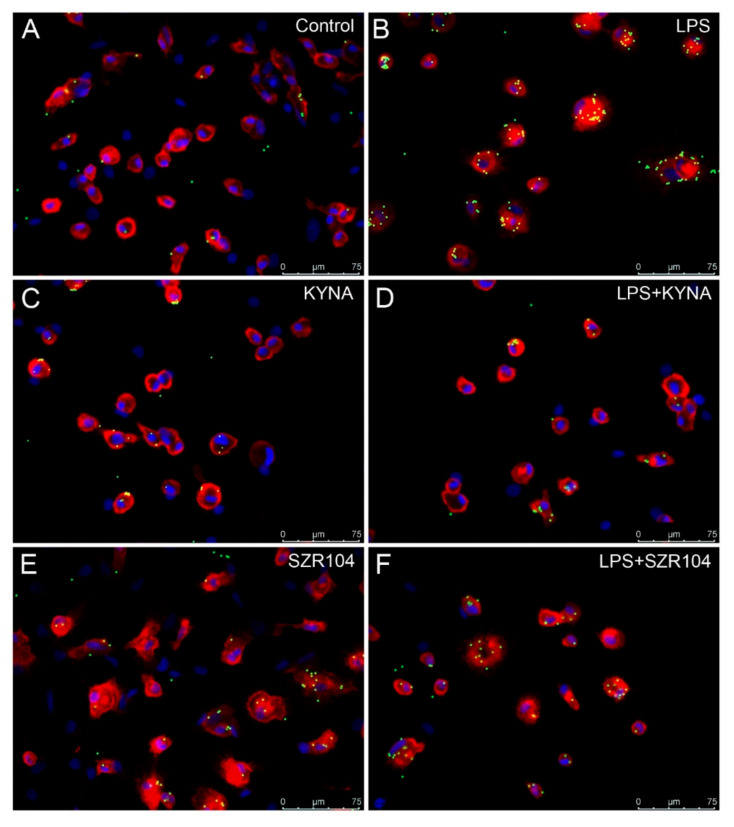
Effects of kynurenic acid (KYNA) and SZR104 on the phagocytic activity of microglial cells in cultures. Pictures showing CD11b/c-immunostained microglia in red, microbeads in green and cell nuclei in blue. (**A**) Unstimulated and untreated control; (**B**) lipopolysaccharide (LPS)-challenged cells; (**C**) KYNA-treated; (**D**) LPS + KYNA-treated; (**E**) SZR104-treated; (**F**) LPS + SZR104-treated. Microglia displayed different phagocytotic activity, as evidenced by the number of phagocytosed microbeads. Scale bar: 75 μm.

**Figure 5:**
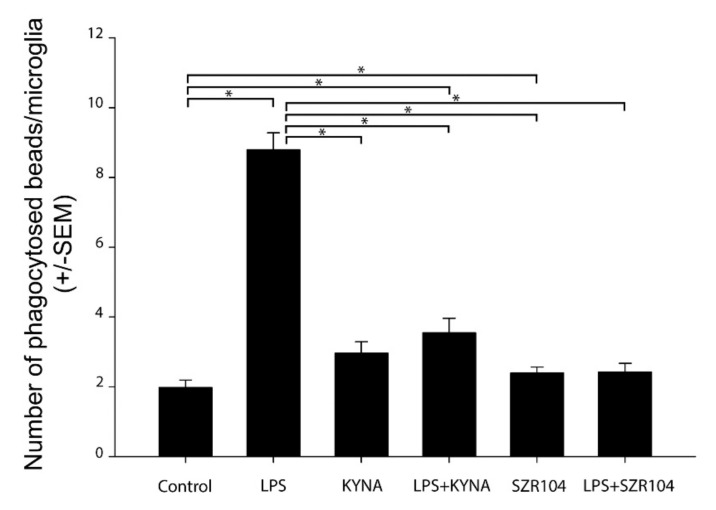
Quantitative analysis of the number of phagocytosed microbeads after the pharmacological treatments demonstrated that kynurenic acid (KYNA) and SZR104 significantly inhibited microglial phagocytosis. The number of phagocytosed beads (*n =* 240; mean ± SEM) was counted in three separate culturing procedures. Data were analyzed with Kruskal–Wallis one-way analysis of variance on ranks. * *p* < 0.05.

**Figure 6:**
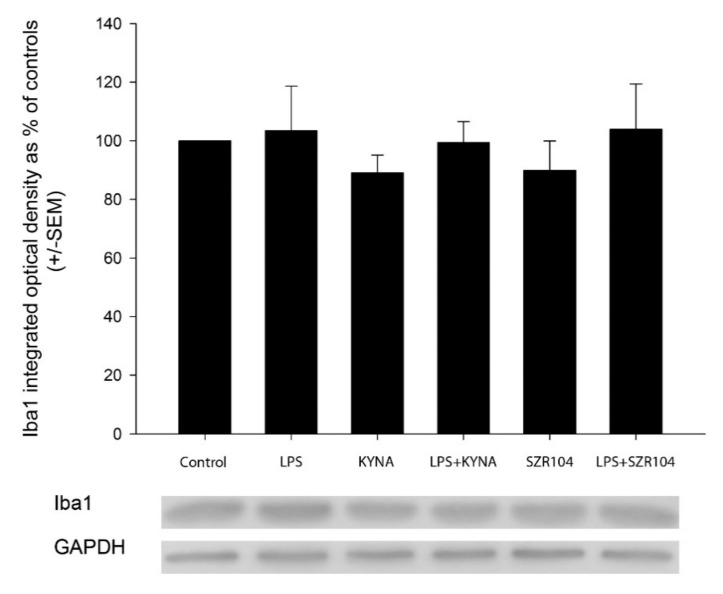
Quantitative Western blot analysis of Iba1 and glyceraldehyde-3-phosphate dehydrogenase (GAPDH) immunoreactivities in microglia cell cultures. Error bars indicate integrated optical density values as percent of controls (*n =* 4; mean ± SEM). Representative Western blot images are shown below the graphs. Data were analyzed with a one-way ANOVA. No statistically significant differences were found.

The treatment of the secondary microglial cultures with LPS increased the phagocytotic activity of the microglia cells significantly (Figure 4 and Figure 5). The unstimulated cultured microglia cells display a basal phagocytotic activity: the number of microbeads in a single cell is regularly two (Figure 4 and Figure 5). Following the LPS challenge, this number rises sharply up to nine per single cell (Figure 4 and Figure 5). Treatment of the cell culture with KYNA and SZR104 before the LPS challenge prevented the increase in the number of phagocytosed microbeads; the phagocytotic activity of the microglia in the presence of KYNA and SZR104 remained at the control level (Figure 4 and Figure 5). This pharmacological effect of the KYNA and SZR104 was significant (Figure 5). Quantitative Western blot analysis of Iba1 expression demonstrated that the Iba1 content of the secondary cultures did not change significantly following the different pharmacological (LPS, KYNA and SZR104) treatments (Figure 6).

## 3. Discussion

The main findings of the experiments are as follows. (1) The KYNA analogue SZR104 did not prevent the symptoms of motor convulsions. At the same time, SZR104 injected before the PILO treatment did not improve the mortality rate of the epileptic animals. (2) The KYNA analogue SZR104 decreased the postepileptic elevated blood IL-6 levels, although the decrease was not significant. (3) The KYNA analogue SZR104 prevented the post-epileptic proliferation of microglia cells in the hippocampus. This effect was statistically significant. (4) The KYNA analogue SZR104 significantly prevented the post-epileptic in vivo reactive morphological transformation of hippocampal microglia. (5) KYNA and the KYNA analogue SZR104 prevented the LPS-induced increase in phagocytotic activity of microglia cells, in vitro. This pharmacological effect was statistically significant. (6) KYNA and SZR104 did not cause alteration of the Iba1 protein expression of in vitro microglia cells.

### 3.1. KYNA is an Endogenous Regulator in the CNS

Tryptophan metabolism is an important process in the central nervous system (CNS) because it is the source of serotonin and melatonin through the methoxyindole pathway [2,7,23]. The other direction of the tryptophan metabolism is the kynurenine pathway (KP), which accounts for more than 90% of tryptophan transformation in the CNS [2,7,23]. One of the key molecules of the KP is L-kynurenine, which results in the formation of other, pharmacologically active compounds, such as KYNA and quinolinic acid (QUIN). KYNA is antagonistic on ionotropic glutamate receptors (iGluRs), especially NMDA- and α-amino-3-hydroxy-5-methyl-4-isoxazole (AMPA)/kainate receptors in micromolar concentrations [2,23], whilst QUIN is neurotoxic because it stimulates the synaptic release of glutamate [1,2,23].

Our previous experiments with iGluR antagonists (dizocilpine, ketamine, amantadine and GYKI52466) proved that iGluR antagonists prevented motor convulsions and decreased epilepsy-related mortality in the experimental animals significantly [6,24]. These effects were not detected in our animal experiments following the SZR104 pretreatment; therefore; we think that SZR104 did not exert its pharmacological effects on iGluRs. These measured microglia effects of KYNA and SZR104 are probably the consequences of the downregulation of inflammation-related genes in microglia cells [25].

### 3.2. Blood IL-6 Level Alterations in Seizure

Post-epileptic elevation of IL-6 in peripheral blood is described in the literature [18]. This elevation of IL-6 1–3 days post-seizure was also measured with RT-PCR and flow cytometry in mice treated with PILO [18]. Blood IL-6 certainly originated from the immune system in these epileptic animals, and some of the IL-6 elevation was certainly due to the secretion of IL-6 by brain microglia [12,18]. SZR104 alone did not cause any alteration in IL-6 blood level and microglia morphology. We think, therefore, that SZR104 acted indirectly in epileptic animals. The animals treated with SZR104 prior to PILO treatment did show some decrease in IL-6 levels. Although this decrease was not significant, we think that the non-significant decrease in IL-6 levels reflected the suppression of microglial inflammation-related genes by SZR104 in our experiments [25]. The inhibition of microglia activation by SZR104 (see next paragraph) supports this assumption. The lack of significance can be explained on the basis of the low number of samples (four samples).

### 3.3. Postepileptic Transformation of Hippocampal Microglia

Microglia cells are activated rapidly following pathological changes in the brain [9]. The expression of neurotransmitter receptors on their surface renders them sensitive to neuronal hyperactivity [12,13,14,15]. The receptors initiate the transformation of microglia from resting to activated status by regulating their cytokine secretion [12,13,14,15,18]. This means that in the acute phase of the post-epileptic period, the secretion of IL-6, tumor necrosis factor α (TNFα), interleukin-12 and interleukin-1β will be boosted and the brain cytokines will be secreted primarily by the microglia cells [18]. The stimulation of proinflammatory IL-6 secretion by neuronal hyperactivity was proven in our experiments. Microglia can promote neurodegeneration [10,11,13] but can also be the protector of neurons [11,13]. It is an open question whether these epileptic microglia cells boosted or delayed the neurodegeneration caused by the PILO epilepsy [26]. Further in vivo experiments are necessary in order to determine the exact role of microglia in our epilepsy experiments.

### 3.4. Inhibition of Phagocytosis In Vitro by KYNA and SZR104

Cultured microglia cells stimulated with LPS displayed a large increase in phagocytosis of fluorescent microbeads. This observation is supported by previous data from our laboratory [20,21,22]. The number of internalized microbeads increased more than four-fold in the stimulated cells. The LPS probably stimulated Toll-like receptor 4 (TLR4) and transient receptor potential ankyrin 1, 4 receptors (TRPA1, TRPA4) [27]. These receptors mediated those membrane and cytoplasmic processes which lead to phagocytosis; they increase the Ca^++^ permeability of the membrane (TRPA effect), exert regulatory effects on gene expression (TLR4 effect) and increase the cytoplasmic cation concentrations (TRPA effect). Treatment of the cells with KYNA and SZR104 prevented the increase in phagocytotic activity by LPS. We think that during the in vitro exposure, KYNA and SZR104 were able to prevent the actions of LPS mainly through the repression of TLR4 by the deregulation of inflammation-related genes, consequently preventing phagocytosis [7,25]. It was proven in monocytic cell cultures that KYNA and SZR104 directly facilitated the expression of anti-inflammatory tumor necrosis factor-stimulated gene-6 (*TSG-6*) and, at the same time, attenuated the TNFα production of these cells [25]. These actions could have been exerted through the cytoplasmic aryl hydrocarbon receptor (AHR) [7,8,25]. Experimental evidence suggested that KYNA is an endogenous ligand of AHR [7,8,28,29,30] and inhibited phagocytosis in myeloid cells through AHR signaling [31].

Literature data prove that other tryptophan derivatives and probably also the synthetic analogue SZR104 are ligands of the AHR [28]. The AHR is a cytoplasmic ligand-dependent transcription factor which was proven to exert immunosuppressive and anti-inflammatory effects [7,8,28,29,30]. The AHR complex is present in the mammalian hippocampus in neurons, astrocytes and microglia cells [29]. The AHR participates in several intracellular regulatory processes, from cell differentiation to apoptosis [7,8,28,29,30]. KYNA is known to be the endogenous modulator of the AHR [7,8], although the molecular details of this action are not clear [25,28]. On the basis of the literature [25,28], we think that SZR104 also acted on the AHR [25]. SZR104 was reportedly crossing the BBB [4] because it was applied successfully in PTZ seizures, in vivo [4]. During the 40-min pretreatment period, SZR104 crossed the BBB and acted on microglial AHRs, inhibiting the transformation of microglia into reactive forms and inhibiting the reentry of the cells into the cell cycle during and following the seizure [29,30]. This effect was the result of the genomic AHR pathway [28]. Since the effect of SZR104 was completely independent of the actions of extracellular glutamate on NMDA receptors, it did not influence the seizure symptoms and did not prevent the mortality of the animals. Similarly, in cell culture, KYNA and SZR104 supposedly acted through the AHR pathway and thereby suppressed phagocytosis through the decrease in the expression of TLR-4 on the cell surface because of the inhibition of inflammation-related gene expression [25]. The repression of some cytoskeletal proteins necessary for migration and phagocytosis was also reported [32]; these finally resulted in the strong decrease in phagocytotic activity of LPS-treated cells [31,32]. We certainly need further experiments to clear the possible role of AHRs in the pharmacological action of SZR104.

## 4. Materials and Methods

### 4.1. Kynurenines Used

The following kynurenines were investigated in our experiments [3,4,25] (Table 2).

### 4.2. In Vivo Experiments

Adult (30 g body weight), male NMRI strain mice were used. Animals were housed in standard conditions with dark/light cycle (12–12 h), room temperature 24 °C and humidity 22%, with food and water ad libitum. The animal experiments were approved first by the Faculty Ethical Committee on Animal Experiments (MÁB), University of Szeged (approval No.: 1-74-1/2017.MÁB), and the Government Office of Csongrád County (Department of Food and Animal Health; approval No.: CS/101/3347-2/2018). The animal experiments were conducted according to the European Union Directive 2010/63/EU of the European Parliament and of the Council of 22 September 2010 on the Protection of Animals Used for Scientific Purposes.

The experiments were performed in the morning. Epilepsy was triggered through intraperitoneal (i.p.) injection of 190 mg/kg pilocarpine hydrochloride (PILO). PILO (Sigma-Aldrich St.Louis, MO, USA) was dissolved in sterile physiological saline (supplied by the Central Pharmacy of the Medical Faculty, Szeged, Hungary). Control animals received i.p. physiological saline. SZR104 was dissolved in distilled water and i.p. administered at a dose of 358 mg/kg 40 min before the PILO injection, according to [4]. The groups of experimental animals are shown in Table 2.

Generalized tonic-clonic seizures developed in the PILO- and SZR104 + PILO-injected animals. At 90 min from the time of the PILO injection, the motor convulsions were stopped with 10 mg/kg diazepam (Seduxen^®^Richter, Budapest, Hungary) i.p., and the animals were treated with Ringer-lactate solution injected intradermally. The animals were observed for 3–4 h after the injections. Mortality was 30% in PILO- and 40% in PILO + SZR104-injected animals. One day (24 h) after the treatments, the surviving animals were anesthetized with halothane (Sigma-Aldrich, St. Louis, MO, USA), the left cardiac ventricle was canulated and the animals were perfused through the heart and ascending aorta with 50 mL cold 4% paraformaldehyde in 0.1 M phosphate-buffered saline (PBS; pH 7.4). Brains were postfixed in the fixative for 24 h, at 4 °C, cryoprotected in 30% sucrose in PBS at room temperature and sectioned on a freezing microtome (Reichert-Jung, Cryocut 1800, Vienna, Austria) in coronal planes at 25-µm thickness between the coronal levels 67 and 79, according to Allen’s Mouse Brain Atlas (www.brain-map.org).

### 4.3. Analysis of Blood Samples

Samples of blood (300–500 μL) were taken from the right ventricle of the heart of the halothane-anesthetized animals with a sterile tuberculin syringe. The blood samples were centrifuged at 3000× *g* for 10 min and then the serum layers were collected and stored at −20 °C until measurement. IL-6 concentrations were determined using the LEGEND MAX^TM^ Elisa kit (BioLegend Inc., San Diego, CA, USA), according to the instructions of the manufacturer. The sensitivity of the assay was 2 pg/mL. The intraassay coefficient of variation was <5.7%, and the interassay coefficient of variation was <10.7%. Statistical analysis of ELISA data was done using R Studio for Windows (version 1.3.1073). Data are expressed as mean ± SEM, differences were calculated with an ANOVA and a post hoc Bonferroni’s test was performed for significance.

### 4.4. Iba1 Immunohistochemistry

The brain sections were processed free-floating, according to the following protocol. Endogenous peroxidase activity was blocked with 3% H_2_O_2_ for 15 min. Non-specific binding sites were blocked with 20% normal pig serum (NPS; diluted: 1/10; Abcam, Cambridge, UK) and tissue permeability was enhanced by using 1% Triton X-100 in the blocking solution. The sections were incubated overnight at room temperature with the primary rabbit anti-Iba1 antibody (1/8000, Fujifilm Wako Chemicals Europe GmbH, Neuss, Germany). Biotinylated secondary antibodies (1:400, Jackson Immuno Research, West Grove, PA, USA) were used for 1 h at room temperature and the signal was detected with peroxidase-labeled streptavidin (1/6000, Rockland Immunochemicals Incorporation, Limerick, PA, USA). All incubations were done in plastic vials with continuous agitation. The sites of the immunoreaction were visualized with diaminobenzidine tetrahydrochloride (DAB) + H_2_O_2_. Sections were mounted on microscope slides, air-dried, dehydrated and coverslipped with DPX (Merck KGaA, Darmstadt, Germany). Chemicals other than antibodies were purchased from Sigma-Aldrich (St. Louis, MO, USA).

### 4.5. Evaluation of the Immunohistochemical Data

The stained sections were scanned with a Slide scanner (Mirax Midi, 3DHistech Ltd., Budapest, Hungary) equipped with a Pannoramic Viewer 1.15.4 program (3DHistech Ltd., Budapest, Hungary). The digitized sections were analyzed using Image Pro Plus 4.5 morphometry software (Media Cybernetics, Silver Spring, MD, USA). Four sections were analyzed from every animal. Using the digital image, the hippocampal formation (subiculum, hippocampus and dentate gyrus) was manually outlined as the area of interest (AOI). The threshold was determined in such a way that the counting program could equally recognize Iba1 immunoreactive microglial cells. The software counted the number of the immunostained cells in the entire AOI (i.e., in every layer of the hippocampal formation). The number of microglia cells in the AOI was normalized to one square millimeter. The surface area of the single microglia cells was also measured with the help of Image Pro Plus 4.5 software (Media Cybernetics, Silver Spring, MD, USA). Single microglia cells were selected from the digital images with 3000× magnification, their contours were manually outlined and their area was measured. The selection and the measurements were relatively simple because of the low staining background and the strong Iba1 immunostaining of the microglial cells. Ten microglia cells were selected from the hippocampi of each experimental group. These cell area measurements were expressed as square micrometer values. Data are expressed as mean ± SEM, differences were calculated with a one-way ANOVA and a post hoc Bonferroni’s test was performed (*p* ≤ 0.05 was significant). GraphPad Prism8 (version 8.4.3) software (GraphPad Software, LLC, San Diego, CA, USA) was used to statistically analyze the results of the immunohistochemical measurements.

### 4.6. Cell Culture Experiments

Microglial-enriched cell cultures were prepared from primary cortical cell cultures. Forebrain cerebrocortical tissue samples were obtained from newborn Sprague Dawley rats. The animals were decapitated, the cerebral cortex was removed, minced with scissors, incubated for 10 min at 37 °C in Dulbecco’s Modified Eagle’s Medium (DMEM; Invitrogen, Carlsbad, CA, USA, containing 1 g/L D-glucose, 110 mg/L Na-pyruvate, 4 mM L-glutamine, 3.7 g/L NaHCO_3_, 10,000 U/mL penicillin G, 10 mg/mL streptomycin sulfate and 25 μg/mL amphotericin B) supplemented with 0.25% trypsin (Invitrogen, Carlsbad, CA, USA) and then centrifuged at 1000× *g* for 10 min at room temperature (RT). The pellet was resuspended, washed twice in 5 mL DMEM containing 10% heat-inactivated bovine serum (Invitrogen, Carlsbad, CA, USA) and centrifuged for 10 min at 1000× *g* at RT. The final pellet was resuspended in 2 mL of the same solution and passed through a sterile filter (100-µm pore size; Greiner Bio-One Hungary Kft., Mosonmagyaróvár, Hungary) to eliminate tissue fragments that resisted dissociation. The filtered suspension was resuspended in 2 mL of the same solution, after which the cells were counted and plated in the same medium either on poly-L-lysine-coated culture flasks (75 cm^2^, 10 × 10^6^ cell/flask) or coverslips (2 × 10^5^ cells/coverslip) and incubated at 37 °C in a humidified air atmosphere supplemented with 5% CO_2_. The DMEM containing 10% bovine serum (BSA) was changed the next day, and then, every 3 days. Secondary microglial cells were subcloned from mixed primary cultures maintained in a poly-L-lysine-coated culture flask by shaking the cultures at 120 rpm in a platform shaker for 20 min at 37 °C. Microglia from the supernatant were collected by centrifugation at 3000× *g* for 8 min at 4 °C and resuspended in 2 mL DMEM containing 10% bovine serum. The cells were seeded at a density of 10^6^ cells/Petri dish for Western blots or 2 × 10^5^ cells/coverslip/Petri dish for immunocytochemistry and phagocytosis assay and cultured in DMEM containing 10% BSA in a humidified atmosphere supplemented with 5% CO_2_ at 37 °C for 7 days (subDIV7). The DMEM containing 10% BSA was changed the next day, and then, every 3 days. On the sixth day of subcloning (subDIV6), the medium was replaced and the microglial cells were treated for 24 h with either LPS (20 ng/mL final concentration, dissolved in DMEM), KYNA (1 µM) or SZR104 (1 µM final concentration) alone or with a combination of LPS + KYNA and LPS + SZR104 and the effects were compared in functional tests (phagocytosis, Iba1 protein synthesis). The LPS treatment served as an inflammation challenge [14]. For the measurement of phagocytic activity, phagocytosed microbeads were counted in the microglia of the secondary cultures. Cultures treated with fluorescently labeled microbeads were incubated for 60 min at 37 °C. The cells were fixed in 4% formaldehyde in 0.05 M PBS (pH 7.4, RT) for 5 min and rinsed in 0.05 M PBS for 3 × 5 min. The cells were first incubated with 0.05 M PBS containing 5% normal goat serum (Sigma-Aldrich, St. Louis, MO, USA) and 0.3% Triton X-100 for 30 min at 37 °C, then treated with mouse anti-CD11b/c antibody (1/200 Invitrogen Carlsbad, CA, USA) in 0.05 M PBS containing 1% BSA solution overnight at 4 °C. After washing at RT (3 × 5 min in 0.05 M PBS), cells were incubated with Alexa Fluor 568 goat anti-mouse immunoglobulin G (IgG; Invitrogen, Carlsbad, CA, USA) diluted (1/1000) in 1 mg/mL polyvinylpyrrolidone/0.05 M PBS containing 1 mg/mL polyvinylpyrrolidone in the dark for 2 h, at RT. The coverslips were then rinsed twice in 1 mg/mL polyvinylpyrrolidone/0.05 M PBS, then in distilled water, and were then dried and mounted on microscope slides covered with Prolong Diamond Antifade with 4′,6-Diamine-2′-phenylindole dihydrochloride (DAPI; Thermo Fisher, Waltham, MA, USA).

### 4.7. In Vitro Phagocytosis Assay

The fluid-phase phagocytotic capacity of the microglial cells was determined via the uptake of fluorescent microspheres (2-μm diameter; Sigma-Aldrich, St.Louis, MO, USA). The cells were plated on coverslips in Petri dishes at a density of 200,000 cells/coverslip in 2 mL DMEM containing 10% heat-inactivated bovine serum and cultured for 7 days (subDIV7); then, 2 μL 2.5% aqueous suspension of fluorescent microspheres was added and the secondary culture was incubated for 60 min at 37 °C. The cells were washed five times in 0.05 M PBS, fixed in 0.05 M PBS containing 4% formaldehyde, and CD11b/c-immunocytochemistry was performed. The coverslips were mounted in Prolong Diamond Antifade with DAPI (Thermo Fisher, Waltham, MA, USA) and the fluorescent microbeads were counted under the microscope with 20× and 40× objectives—100 random fields with a total of 1690 bead-labeled cells were counted and the number of phagocytosed microbeads (mean ± SEM) was analyzed. Statistical comparisons were made using SigmaPlot (v. 12.3, Systat Software Inc., Chicago, IL, USA) and data were analyzed with a Kruskal–Wallis one-way analysis of variance on ranks, followed by Dunn’s method for pairwise multiple comparison procedures for statistically significant differences between the groups.

### 4.8. Western Blot Analysis

Cultured cells were collected with a rubber policeman, homogenized in 50 mM TrisHCl (pH 7.5) containing 150 mM NaCl, 0.1% Nonidet P40, 0.1% cholic acid, 2 μg/mL leupeptin, 1 μg/mL pepstatin, 2 mM phenylmethylsulfonyl fluoride and 2 mM EDTA, incubated on ice for 30 min and centrifuged at 10,000× *g* for 10 min. The pellet was discarded, and the protein concentration of the supernatant was determined [33]. For the Western blot analysis of Iba1 immunoreactivity, 10 μg protein was separated on sodium dodecyl sulfate (SDS)-polyacrylamide gel, transferred onto Hybond-ECL nitrocellulose membrane (Amersham Biosciences, Little Chalfont, UK), blocked for 1 h in 5% non-fat dry milk in Tris-buffered saline (TBS) containing 0.1% Tween 20 and incubated with either a rabbit anti-Iba1 polyclonal antibody (dilution 1/1000; Fujifilm Wako GmbH, Neuss, Germany) or a mouse anti-glyceraldehyde-3-phosphate dehydrogenase (GAPDH) monoclonal antibody (clone GAPDH-71.1; 1/20,000 final dilution; Sigma-Aldrich, St.Louis, MO, USA). Non-specifically bound or excess antibody was removed with 5 × 5 min rinses in 0.1 M TBS containing 0.1% Tween 20. Membranes were then incubated for 1 h with either peroxidase-conjugated goat anti-rabbit or peroxidase-conjugated rabbit anti-mouse antibody (dilution 1/2000; Sigma-Aldrich, St. Louis, MO, USA) and washed five times in 0.1% TBS–Tween 20. The enhanced chemiluminescence method (ECL plus Western blotting detection reagents; Amersham Biosciences, Little Chalfont, UK) was used to reveal immunoreactive bands, according to the manufacturer’s instructions.

### 4.9. Image Analysis

Grayscale digital images of the Western blots were acquired by scanning the autoradiographic films with a desktop scanner (Epson Perfection V750 Pro; Seiko Epson Corp., Nagano, Japan). Images were scanned and processed at identical settings to allow comparisons of the blot results from different samples. The densities of immunoreactive lanes equally loaded with protein were quantified and presented as percent of controls. A one-way ANOVA (SigmaPlot, v.12.3, Systat Software Inc., Chicago, IL, USA) was used for statistical comparisons. Values as percent of controls are presented as the mean ± SEM from at least four immunoblots, each representing an independent experiment.

## 5. Conclusions

We investigated the possible pharmacological effects of the synthetic KYNA analogue SZR104. SZR104 crosses the BBB [4]. In the present experiments, SZR104 did not prevent behavioral convulsions and did not decrease the mortality of epileptic mice subjected to systemic pilocarpine treatment. On the other hand, SZR104 significantly decreased the number of microglia cells in the epileptic hippocampus and prevented microglia proliferation. Furthermore, SZR104 significantly decreased the size of individual microglia cells in the epileptic hippocampus and prevented microglia activation. Finally, SZR104 and KYNA significantly decreased microglial phagocytosis in vitro.

These experimental results were explained on the basis of data from the literature and the following hypothetic conclusions were drawn:

1. Although it was previously supposed that KYNA analogues act as iGluR antagonists [1,2,3,4,23], the present SZR104 action mechanism was not connected to NMDA receptors. SZR104 was acting through non-glutamatergic molecular mechanisms.
2. We supposed that SZR104 actions were exerted through the AHR complex [25]. KYNA and the synthetic analogue SZR104 are ligands of the AHR [28].
3. The action on AHRs could explain the inhibitory effect of SZR104 on microglia activation and proliferation. The phenotype change of the microglia could be the consequence of the genomic actions of the AHR complex [28], through a molecular scenario including the decrease in TNFα secretion and increasing tumor necrosis factor-stimulated gene-6 (*TSG-6*) expression [25]. The depletion of TNFα may affect multi-protein complex containing mammalian target of rapamycin (mTOR) protein (mTORC1) activity and the nuclear translocation of mTOR [34]. The depletion of TNFα and mTORC1 activation may arrest the cell cycle in the late G1 phase through metabolic checkpoints [35], thereby preventing microglia proliferation.
4. The inhibition of microglia phagocytosis by SZR104 and KYNA could also utilize AHR pathways, causing the decrease in TLR-4 expression on the cell surface [28], the induction of cytoskeletal changes [32] or the deregulation of the nuclear factor kappa-light-chain-enhances of activated B cells (NF-κB) pathway [28].

## Figures and Tables

**Table 1:** Animals used in pharmacological-immunohistochemical experiments and blood sampling for interleukin-6 (IL-6) ELISA measurement. The differences in surviving animal numbers reflected the increased mortality of the SZR104 + PILO-treated animals (see Results).

Pharmacological Treatment	Number of Surviving Animals	Experimental Procedures
Control animals: 0.9% NaCl i.p. injection	12	Blood samples (8 animals). Immunohistochemistry (4 animals).
Pilocarpine-treated: 190 mg/kg PILO, i.p. injection	21	Blood samples (18 animals). Immunohistochemistry (4 animals).
SZR104 solution i.p. (358 mg/kg)	12	Blood samples (8 animals). Immunohistochemistry (4 animals).
SZR104 (358 mg/kg) and PILO (190 mg/kg) i.p. injections	8	Blood samples (4 animals). Immunohistochemistry (4 animals).

**Table 2:** Molecular structure, chemical name, empirical formula and molecular weight of KYNA and its analogue SZR104.

Code	Molecular Structure	Chemical Name	Empirical Formula and Molecular Weight
KYNA	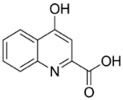	4-hydroxyquinolin-2-carboxylic acid	C_10_H_7_NO_3_ 189.17
SZR104	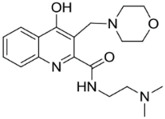	*N*-(2-(dimethylamino)ethyl)-3-(morpholinomethyl)-4-hydroxyquinoline-2-carboxamide	C_19_H_26_N_4_O_3_ 358.43

## Abbreviations

AHR	Aryl hydrocarbon receptor
AMPA	α-amino-3-hydroxy-5-methyl-4-isoxazole
ANOVA	Analysis of variance
AOI	Area of interest
BBB	Blood–brain barrier
CNS	Central nervous system
ELISA	Enzyme-linked immunosorbent assay
GAPDH	Glyceraldehyde-3-phosphate dehydrogenase
GYKI52466	a 2,3-benzodiazepine, which is non-competitive AMPA receptor antagonist
Iba1	Ionized calcium binding adaptor molecule 1
iGluR	Ionotropic glutamate receptor
IL-6	Interleukin-6
KYNA	Kynurenic acid
KP	Kynurenine pathway
LPS	Lipopolysaccharide, endotoxin
mTOR	Mammalian target of rapamycin
mTORC1	Multi-protein complex containing mTOR protein
NF-κB	Nuclear factor kappa-light-chain-enhances of activated B cells; protein complex regulating cellular responses
NMDA	N-methyl-D-aspartic acid
PILO	Pilocarpine
RT-PCR	Reverse transcription polymerase chain reaction
QUIN	Quinolinic acid
SEM	Standard error of the mean
SZR104	N-(2-(dimethylamino)ethyl)-3-(morpholinomethyl)-4-hydroxyquinoline-2-carboxamide
TLR4	Toll-like receptor4
TNFα	Tumor necrosis factor-alpha
TRPA1,4	Transient receptor potential ankyrin 1,4 receptors
